# Parastomal Gallbladder Herniations: A Systematic Review

**DOI:** 10.7759/cureus.71379

**Published:** 2024-10-13

**Authors:** Siji Olusola, Tobias Jarman, Chetan Parmar, Manikandan Kathirvel

**Affiliations:** 1 Department of General Surgery, Whittington Hospital, London, GBR; 2 Department of Bariatric Surgery, Whittington Health NHS Trust, Whittington, GBR; 3 Department of Hepato-Pancreato-Biliary and Liver Transplantation, Whittington and Royal Free Hospital, London, GBR

**Keywords:** cholecystectomy, cholecystitis, gallbladder, parastomal hernia, stoma

## Abstract

Parastomal hernias (PSHs) are a common complication following stoma placement. The presence of the gallbladder within a PSH is a rare and unusual occurrence, posing a significant management challenge for surgeons once encountered. We conducted a systematic review of the literature to evaluate the management and outcomes of parastomal gallbladder herniations. A comprehensive search was performed across the PubMed, Embase, and Medline databases using the search terms “gallbladder” AND “parastomal hernia.” Additionally, a reference check of the results was conducted to identify further case reports. Eighteen case reports were included in the review. The mean age of the reported cases was 77.2 years, with a female predominance of 88.9% (n = 16). Seventy-seven percent (n = 14) of patients had an original ileal stoma or conduit. The average duration from stoma placement to clinical presentation was 5.8 years and abdominal pain was the most common presenting complaint. CT imaging was the most frequently utilised modality for successful diagnosis, with only two herniations identified during surgical exploration. Notably, 66.6% (n = 12) of patients experienced associated gallbladder complications, including cholecystitis, torsion, incarceration, and even perforation, all of which necessitated cholecystectomy. Cholecystic parastomal herniation is a rare phenomenon that should be considered in differential diagnoses for similar presentations. Currently, no standardized classification or management approach exists. Based on our findings, we propose classifying gallbladder herniations into two categories: simple (without inflammatory sequelae) and complicated (with cholecystitis, gallbladder torsion, incarceration, or perforation). Simple herniations may be managed electively with intraoperative reduction of the gallbladder. In contrast, complicated herniations presenting during acute admissions require emergency surgical intervention, involving a combined cholecystectomy and PSH repair.

## Introduction and background

Stoma placement is a common surgical procedure associated with significant complications, one of which is parastomal hernias (PSHs). According to the European Hernia Society, PSH is defined as an abnormal protrusion of abdominal cavity contents through a defect in the abdominal wall created during the placement of a colostomy, ileostomy, or ileal conduit stoma [1]. The incidence of PSH can reach as high as 56% in patients with colostomies and ileostomies, often leading to symptomatic presentations and the need for surgical repair [2,3]. A rare but documented occurrence is the herniation of the gallbladder into a PSH, predominantly affecting elderly females [4]. To better understand the management and outcomes of this condition, we conducted a systematic review of the literature.

## Review

Methods

We conducted a systematic literature search across the PubMed, Embase, and Medline databases using the key terms “gallbladder” AND “parastomal hernia.” This research was performed in accordance with the Preferred Reporting Items for Systematic reviews and Meta-Analyses (PRISMA) guidelines [5]. Given the rarity of this condition, we included case reports in our review. Specifically, we considered case reports that documented the gallbladder as a component of a PSH, encompassing all genders and ethnicities.

Exclusion criteria consisted of articles not published in English, those reporting gallbladder herniation into a non-PSH, and those detailing other visceral components within a PSH. Additionally, we analyzed all references from the articles found to identify any further reported cases. The results were independently reviewed by two evaluators, and we subsequently examined patient demographics, stoma type, clinical presentation, diagnostic modalities, laboratory investigations, complications, treatment approaches, and outcomes.

Results

**Figure 1:**
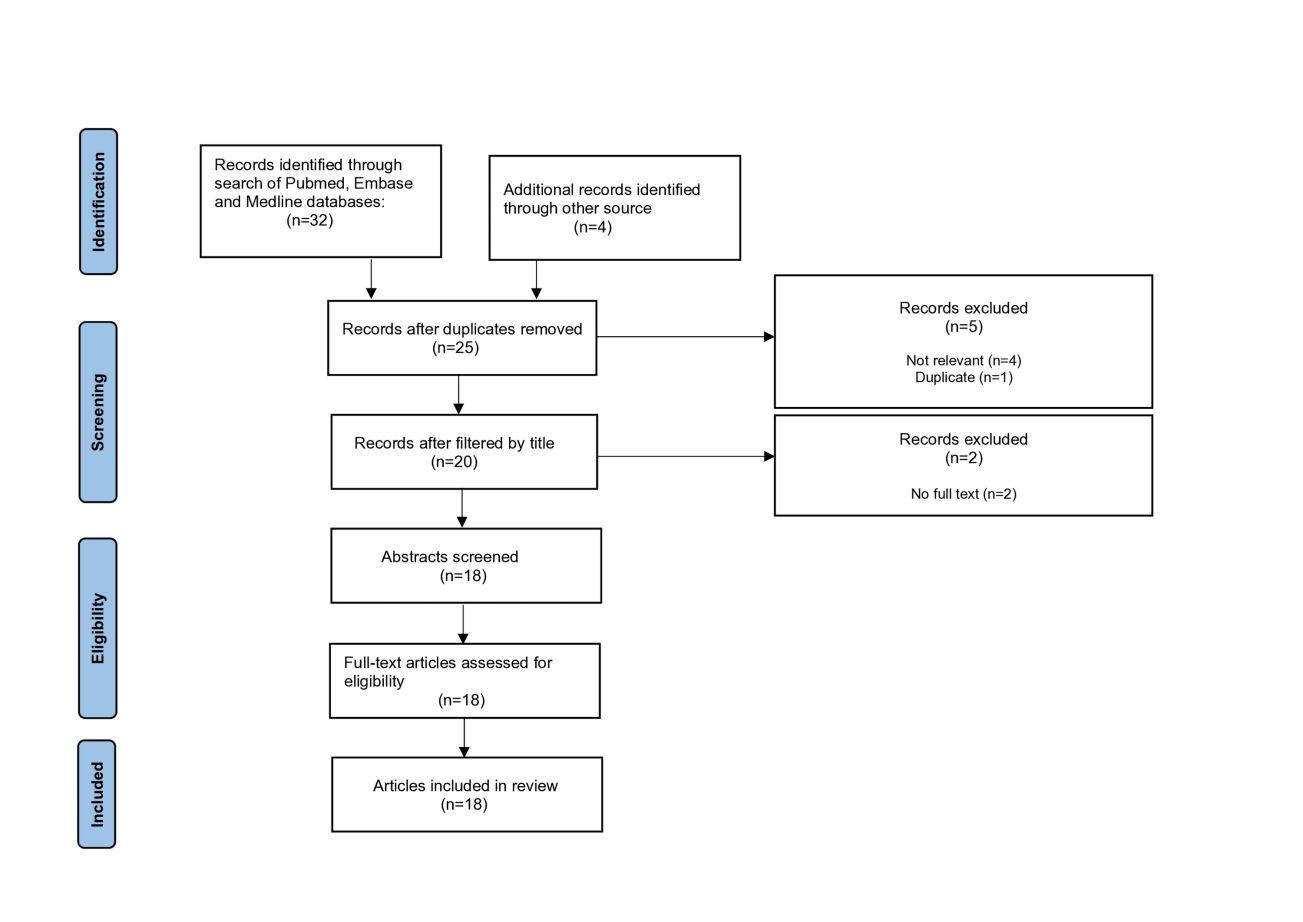
PRISMA flow chart PRISMA, Preferred Reporting Items for Systematic reviews and Meta-Analyses

Our literature search yielded an initial 36 records (Figure 1). After removing duplicates and clearly inappropriate records, 16 were excluded. An additional two records were excluded due to the unavailability of full text. This resulted in a total of 18 papers [6-23] included for final review, the details of which are summarized in Table 1.

**Table 1:** Summary of previously documented cases of parastomal gallbladder herniations ALP, alkaline phosphatase; ALT, alanine aminotransferase; AST, aspartate aminotransferase; CBD, common bile duct; PSH, parastomal hernia; WCC, white cell count

Authors	Age (years)	Sex (M/F)	Previous operation and stoma type	Clinical presentation	Laboratory findings	Diagnostic modality	Complications	Management
St. Peter and Heppell [6]	73	F	Ileal conduit	Acute incarceration of PSH	WCC 19 × 10^9^/L (77% neutrophils); total bilirubin concentration of 1.7 mg/dL	Surgical exploration	Inflamed gallbladder visualized in theaters	Cholecystectomy with local hernia repair
Garcia et al. [7]	63	F	Colectomy with end transverse colostomy	Abdominal pain, nausea, and anorexia with increased colostomy output	Reported as unremarkable	CT abdomen and pelvis	-	Conservative approach with manual reduction of PSH and bowel rest
Rashid et al. [8]	74	F	Total colectomy with end ileostomy formation	Features of small bowel obstruction	WCC 15.5 × 10^9^/L with neutrophilia and raised bilirubin	Surgical exploration	Large, incarcerated gallbladder with signs of dusky constriction seen in theaters	Open cholecystectomy with pre-peritoneal mesh repair of PSH
Rosenblum et al. [9]	76	M	Colectomy with ileostomy formation	Abdominal pain	WCC 18.9 × 10^9^/L	CT abdomen and pelvis without contrast	Gallbladder torsion seen on CT; necrotic gallbladder with a 360-degree twist seen in theaters	Open cholecystectomy
To et al. [10]	85	F	Total cystectomy with formation of a right iliac fossa ileal conduit	Abdominal pain and nausea	Reported as having an elevated WCC	CT abdomen with oral gastrografin contrast	Edematous gallbladder; cholecystitis of incarcerated gallbladder visualized in theaters	Open cholecystectomy with no closure of the parastomal defect
Gomez-Artacho et al. [11]	50	F	Anterior resection of the rectum with a diverting lateral colostomy	Abdominal pain, vomiting, and stomal swelling	Reported as having elevated acute-phase reactants	CT scan	Gallbladder hydrops with incipient signs of acute cholecystitis	Right subcostal laparotomy with cholecystectomy and primary hernia repair
Frankl et al. [12]	88	F	Sigmoid colectomy with a transverse loop colostomy	Fevers and abdominal pain	Reported as having an elevated WCC and mildly elevated ALP, AST, and ALT	CT abdomen and pelvis	Chronically inflamed gallbladder visualized on CT; positive blood cultures for *Klebsiella pneumoniae *presumably secondary to cholecystitis	Given the patient’s age and functional status, conservative management was employed, which included manual reduction of the PSH and antibiotic therapy
Bakshi et al. [13]	89	M	Hartmann’s procedure with later reversal of the colostomy with a diverting loop ileostomy	No stoma output, nausea, frequent burping, and burning sensation around the stoma site	WCC 8.1 × 10^9^/L (82.4% neutrophils)	CT abdomen and pelvis	Acute cholecystitis causing a small bowel obstruction on CT	Conservative management including antibiotic therapy and nasogastric decompression due to concomitant small bowel obstruction
Brown et al. [14]	63	F	Right upper quadrant end transverse colostomy	Large peristomal bulge and abdominal bloating	-	CT abdomen and pelvis	Incarceration of the small bowel and gallbladder	Colostomy takedown with completion sigmoid colectomy, cholecystectomy, and abdominal reconstruction with on-lay bioprosthetic mesh
Rogers et al. [15]	75	F	Subtotal colectomy and formation of end ileostomy	Abdominal pain with intermittent nausea and vomiting	-	CT abdomen and pelvis	-	Intraoperative gallbladder reduction with PSH repair using onlay mesh
Guo et al. [16]	63	F	Open subtotal colectomy and end ileostomy creation	Epigastric discomfort and vomiting	Raised serum amylase of 156 U/L and reported as having a neutrophilia	CT abdomen	Pancreatitis, dilated CBD, and acute cholecystitis on CT	Initially managed conservatively; readmitted one week later and received midline laparotomy, intraoperative cholangiogram, cholecystectomy, and PSH repair using a biological permacol mesh
Moeckli et al. [17]	69	F	Initial right hemicolectomy and ileotransverse colostomy, with a further creation of a colostomy and terminal ileostomy	Parastomal tenderness and pain	CRP 22 mg/l, procalcitonin 0.12 ug/l, and WCC 18’560/ul with neutrophilic shift	CT abdomen and pelvis	-	Elective laparotomy with retrograde cholecystectomy; ostomy reversed with side-to-side ileocolic anastomosis; parastomal defect closed with continuous nonabsorbable suture
Linn and Lim [18]	63	F	Small bowel resection with end ileostomy creation	First presentation: fever and abdominal pain; second presentation eight days later: abdominal pain and vomiting	First presentation: reported as unremarkable; second presentation: mildly elevated amylase of 100 U/L and lipase of 105 U/L	CT abdomen and pelvis	Groove pancreatitis was seen on the first CT scan; acute cholecystitis was seen on the second CT eight days later	Exploratory laparotomy, cholecystectomy, and PSH repair
Smarda et al. [19]	65	F	Low anterior resection and prophylactic loop ileostomy	N/A	N/A	Incidental finding on CT abdominal imaging	-	Intraoperative gallbladder reduction, PSH neck sutured, and end colostomy formation
Crane et al. [20]	87	F	Colectomy and ileostomy creation	Parastomal pain, abdominal distention, and no stoma output	Reported as having a raised CRP	CT abdomen	Gallbladder perforation	Small bowel resection with ileostomy refashioning and an open cholecystectomy via a Kocher incision; suture repair over the hernia defect
Urbonas and Boyce [21]	71	F	Panproctocolectomy with end ileostomy for Crohn’s disease 42 years prior	Parastomal and upper abdominal pain; nausea and bilious vomiting	Deranged LFTs: ALT 273 U/L, ALP 174 U/L, and bilirubin 21 umol/L	CT abdomen	Coincidental transient biliary obstruction secondary to sludge in the CBD	Conservative approach with analgesia and manual reduction of PSH
Seang et al. [22]	87	F	Open cystectomy, total hysterectomy, and formation of ileal conduit	Abdominal pain, nausea, and PSH distention	WCC 14.5 × 10^9^/L, CRP 14 mg/L, lipase 1,349 U/L, and bilirubin 9 umol/L; reported as having deranged liver enzymes	CT abdomen	Perforated gallbladder cholecystitis with subsequent biliary peritonitis	Emergency laparotomy with cholecystectomy with PSH sac lavage
Pinnock et al. [23]	59	F	Exploratory laparotomy with completion proctectomy and end ileostomy	Abdominal pain, nausea and vomiting	WCC 11.45 × 10^9^/L	Non-contrast CT abdomen and pelvis	-	Diagnostic laparoscopy revealed a spontaneous reduction of the gallbladder had taken place

Among the previously reported cases, there was a female predominance of 88.9% (n = 16) and an average age of 72.2 years (range: 50-89). Notably, 77.8% (n = 14) of these cases involved an ileal stoma or conduit, likely due to the close proximity of the gallbladder to ileal stomas in the right hemiabdomen. For all reported cases with available data, the average duration from stoma placement to clinical presentation was 5.8 years. Upon becoming symptomatic, patients invariably presented with abdominal pain, nausea, and/or vomiting, accompanied by parastomal tenderness. Interestingly, only one patient was asymptomatic; in this case, the herniation was discovered incidentally during a routine preoperative CT scan.

In most cases, the most common laboratory finding was an elevated white cell count or neutrophilia. The majority of herniations were confirmed via CT imaging, with only two identified during surgical exploration. Twelve of the 18 cases were further complicated by serious sequelae, including acute cholecystitis, gallbladder torsion, incarceration with necrosis, and even perforation with peritonitis. All patients listed in Table 1 with associated gallbladder complications who were deemed suitable surgical candidates underwent cholecystectomy. Intraoperative gallbladder reduction was performed for patients with a non-inflamed gallbladder. Only two patients who underwent surgery did not receive PSH repair. Notably, one patient represented with a recurrence of their PSH after originally receiving mesh repair two years earlier. The stoma was later re-positioned.

Four of the 18 patients were successfully managed with a conservative, nonoperative approach and were reported to be well during follow-up. Two patients, initially managed conservatively, ultimately required surgical intervention after re-presenting with worsening symptoms seven and eight days later, respectively [16,18]. No mortalities were reported.

Discussion

PSH is a common complication following ostomy creation, with an incidence rate of up to 56% in patients with colostomies [3]. Risk factors for PSH include poor nutritional status, increased intra-abdominal pressure, and corticosteroid therapy, particularly in patients with chronic obstructive pulmonary disease [4]. The surgical creation of a stoma inherently weakens the abdominal wall, predisposing patients to herniation [24]. Although the use of prophylactic mesh during the initial stoma creation may help prevent PSH formation, careful management of infection risk is essential [25].

Gallbladder herniation into a PSH is exceedingly rare, with only 18 cases documented in the medical literature (Table 1). In addition to PSHs, the gallbladder has been reported to herniate into epigastric, incisional, and even inguinal hernias [26,27]. This condition primarily affects elderly females, and its rarity can be attributed to anatomical and physiological factors associated with ageing, such as decreased connective tissue elasticity and a mobile gallbladder due to a long mesentery or liver atrophy [28,29]. These factors may result in gallbladder visceroptosis, thereby increasing the risk of herniation.

Diagnosis is most reliably confirmed through imaging studies, particularly CT scanning, which can demonstrate the absence of the gallbladder from its anatomical position and its presence within the hernial sac [13,15]. CT imaging is preferred for its ability to provide detailed anatomical information, which is crucial for surgical planning and appears to be the optimal diagnostic modality of choice [7].

Management Strategies

Management of parastomal gallbladder herniation can be conservative or surgical, depending on the patient’s overall health and symptom severity. Conservative management involves ward-based strategies, including manual hernia reduction, antibiotic therapy, and close monitoring, making it suitable for frail patients or those without significant symptoms. Bakshi et al. indicated that they refrained from attempting manual reduction due to acute cholecystitis causing friability of the gallbladder, increasing the risk of rupture [13]. Conversely, Frank et al., whose patient was presumed to have cholecystitis, managed to reduce their hernia and complete successful conservative management [12].

Surgical management typically entails hernia repair and cholecystectomy, particularly when the herniation is symptomatic or complicated by conditions such as cholecystitis or gallstones [12,15]. This intervention is often necessary to prevent serious complications, including gallbladder incarceration or bowel obstruction [9,13]. Compression of the cystic duct and cystic artery within the hernial ring can lead to these inflammatory sequelae [27]. To et al. suggested that strangulation of the gallbladder at its narrow neck can indeed predispose to the development of cholecystitis [10]. Rosenblum et al., whose patient ultimately developed a gangrenous cholecystitis, attributed their abnormal findings (elongation and deformity of the gallbladder neck) to the ectopic location and abnormal orientation of the gallbladder [9]. Surgical management will vary based on individual circumstances; for instance, cholecystectomy was electively performed in one patient without radiographic evidence of cholecystitis due to the presence of a large gallstone in the gallbladder fundus [17].

Hernia repair can utilize mesh, although material choice must account for the risk of infection, especially in contaminated settings [4,30]. Recent advancements in mesh technology, including bio-prosthetic and lightweight meshes, show promising results with reduced infection risks [31]. Combining cholecystectomy with mesh repair may increase the risk of mesh infection. To et al. minimized intraoperative contamination by decompressing the gallbladder using a wide-bore needle, which served as a method of source control [10]. Alternatively, suture repair of the hernial defect can be performed, although this method is associated with higher recurrence rates [32]. Overall, the surgical management of PSHs remains a subject of debate, and the presence of rare complications, such as gallbladder herniation, complicates this for surgeons.

The timing of surgical intervention is crucial. Only one patient in Table 1, as reported by Moeckli et al., was managed electively [17]. The remaining patients underwent surgery during their acute admission due to the necessity and severity of their respective presentation. In the one elective case, three factors supported this approach: the patient was a suitable surgical candidate, there was no radiographic evidence of inflammatory sequelae, and initial conservative management led to significant symptom improvement. Had the patient’s symptoms not resolved or worsened, surgical management during the acute admission would have likely been required.

Gallbladder herniation into a PSH is a rare but significant condition that demands careful diagnosis and management. Treatment should be guided by clinical presentation, radiological diagnosis, and a multidisciplinary team approach. Currently, no standardized classification or management approach exists. While conservative management may be appropriate for asymptomatic patients, surgical intervention is generally preferred due to the potential for severe complications.

Most patients will likely require surgical intervention. Further research is needed to establish optimal management strategies and preventive measures for PSHs complicated by gallbladder herniation. As summarized in Table 2, we propose classifying gallbladder herniations as either simple (without inflammatory sequelae) or complicated (with cholecystitis, gallbladder torsion, incarceration, and/or perforation).

**Table 2:** Proposed classification system for gallbladder parastomal herniations

Simple gallbladder herniation	Complicated gallbladder herniation
Gallbladder visualized within a hernial defect in the absence of inflammatory sequelae	Gallbladder visualized within a hernial defect with CT and/or operative findings of at least one of: (1) cholecystitis; (2) gallbladder perforation; (3) gallbladder torsion; and (4) gallbladder incarceration

**Figure 2:**
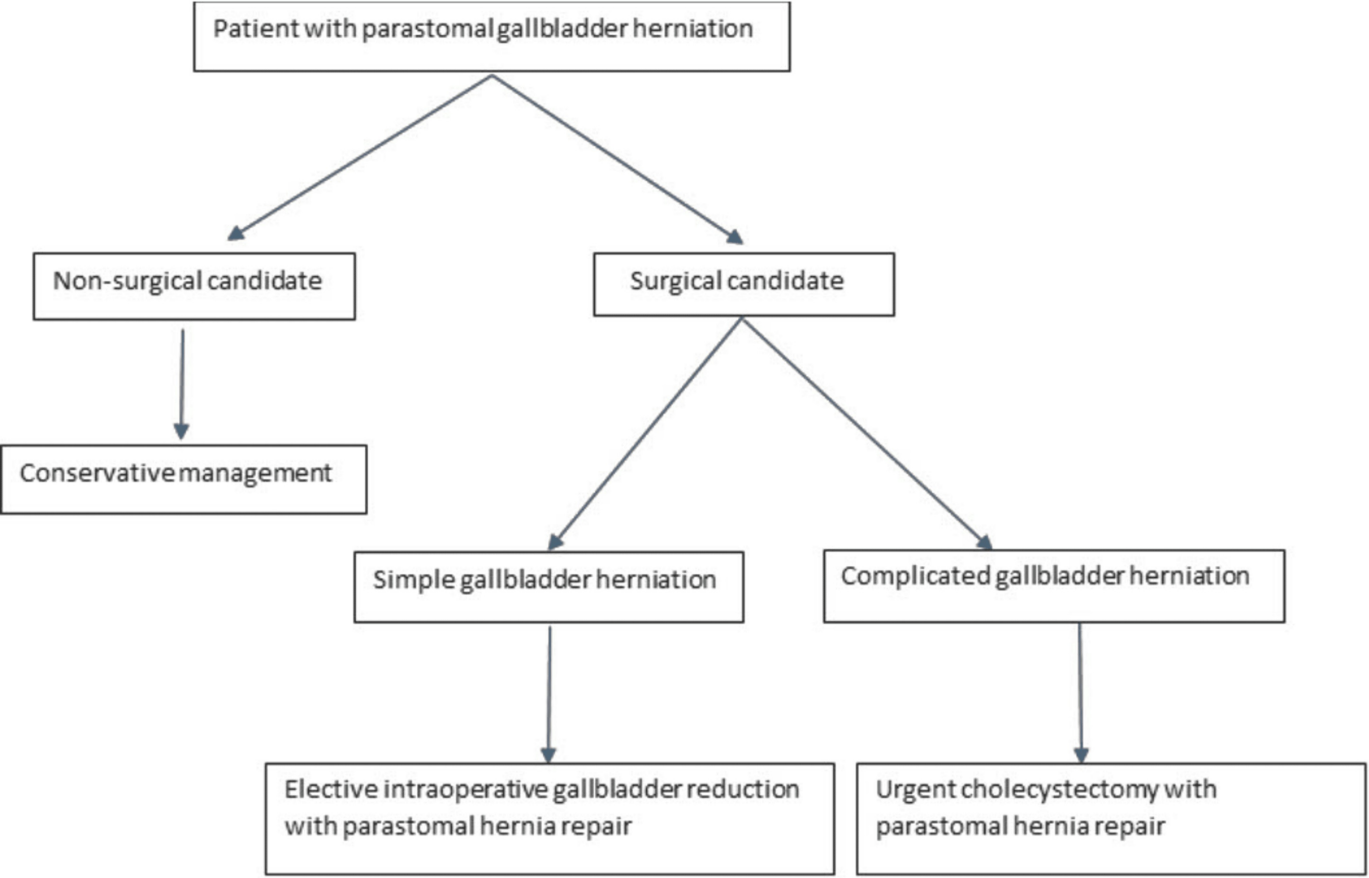
Proposed algorithm for the management of gallbladder parastomal herniations

Simple herniations can be managed electively, with intraoperative reduction of the gallbladder being sufficient. In cases where simple herniations present with non-resolving or worsening symptoms, further evaluation via CT scanning and/or surgical exploration may be warranted. Conversely, complicated herniations encountered during acute admissions necessitate emergency surgical intervention, typically involving a combined cholecystectomy and PSH repair. Figure 2 illustrates a proposed algorithm for the management of parastomal gallbladder herniations.

Limitations

This study has several limitations. Due to the rarity of this condition, we could only include a small number of published single-case reports in our review. Consequently, an element of publication bias is likely present, which may limit the generalizability of our findings. Despite these limitations, this article represents the first systematic review of this rare pathology and aims to enhance its awareness among surgeons.

## Conclusions

Gallbladder herniation into a PSH is a rare yet significant differential diagnosis for patients presenting with acute abdominal pain at a stoma site. Timely diagnosis and appropriate management are essential for achieving favorable outcomes. This systematic review underscores the importance of recognizing this condition and emphasizes the necessity of surgical intervention in managing symptomatic or complicated cases.

## References

[1] Śmietański M, Szczepkowski M, Alexandre JA (2013). European Hernia Society classification of parastomal hernias. Hernia.

[2] Renzulli P, Candinas D (2007). Intestinal stomas--indications, stoma types, surgical technique [Article in German]. Ther Umsch.

[3] Lambrichts DP, de Smet GH, van der Bogt RD (2018). Incidence, risk factors and prevention of stoma site incisional hernias: a systematic review and meta-analysis. Colorectal Dis.

[4] Antoniou SA, Agresta F, Garcia Alamino JM (2018). European Hernia Society guidelines on prevention and treatment of parastomal hernias. Hernia.

[5] Page MJ, McKenzie JE, Bossuyt PM (2021). The PRISMA 2020 statement: an updated guideline for reporting systematic reviews. BMJ.

[6] St. Peter SD, Heppell J (2005). Surgical images: soft tissue. Incarcerated gallbladder in a parastomal hernia. Can J Surg.

[7] Garcia RM, Brody F, Miller J, Ponsky TA (2005). Parastomal herniation of the gallbladder. Hernia.

[8] Rashid M, Abayasekara K, Mitchell E (2009). A case report of an incarcerated gallbladder in a parastomal hernia. Int J Surg.

[9] Rosenblum JK, Dym RJ, Sas N, Rozenblit AM (2013). Gallbladder torsion resulting in gangrenous cholecystitis within a parastomal hernia: findings on unenhanced CT. J Radiol Case Rep.

[10] To H, Brough S, Pande G (2015). Case report and operative management of gallbladder herniation. BMC Surg.

[11] Gomez-Artacho M, Boisset G, Taoum C (2017). Complicated parastomal hernia as a clinical presentation of a gallbladder hydrops. Cir Esp.

[12] Frankl J, Michailidou M, Maegawa F (2017). Parastomal gallbladder hernia in a septic patient. Radiol Case Rep.

[13] Bakshi C, Ruff S, Caliendo F, Agnew J (2017). Acute cholecystitis in a parastomal hernia causing a small bowel obstruction. J Surg Case Rep.

[14] Brown RA, Kann B, Woolridge J (2018). Parastomal hernia with incarcerated gallbladder: a case report. Int J Surg Res Pract.

[15] Rogers P, Lai A, Salama P (2019). Gallbladder complicating a parastomal hernia. J Surg Case Rep.

[16] Guo Y, Lye TJ, Lee ZJ (2020). Gallbladder herniation and cholecystitis within a parastomal hernia: a case report and literature review. Dig Med Res.

[17] Moeckli B, Limani P, Clavien PA, Vonlanthen R (2020). Parastomal gallbladder herniation: a case report and review of the literature. Int J Surg Case Rep.

[18] Linn YL, Lim EK (2023). A very unusual complication of groove pancreatitis. Gastroenterology.

[19] Smarda M, Manes K, Fagkrezos D, Argiropoulos D, Laios K, Triantopoulou C, Maniatis P (2021). Parastomal gallbladder herniation as an incidental preoperative computed tomography finding. Case Rep Radiol.

[20] Crane J, Ari K, Lam S, Lewis M (2021). Perforated gallbladder in a parastomal hernia. BMJ Case Rep.

[21] Urbonas T, Boyce SA (2022). A rare case of parastomal hernia complicated by gallbladder incarceration. ANZ J Surg.

[22] Seang S, Hort A, Gosal PK, Richardson M (2022). A case of perforated cholecystitis into a parastomal hernia. Case Rep Surg.

[23] Pinnock N, Vashi A, Marsh JW, Keita MP, Checovich A (2023). Spontaneous resolution of parastomal gallbladder herniation after attempted surgical intervention: a case report and review of the literature. Cureus.

[24] Helgstrand F, Rosenberg J, Kehlet H, Jorgensen LN, Wara P, Bisgaard T (2013). Risk of morbidity, mortality, and recurrence after parastomal hernia repair: a nationwide study. Dis Colon Rectum.

[25] Lupinacci RM, Gizard AS, Rivkine E (2014). Use of a bioprosthetic mesh in complex hernia repair: early results from a French multicenter pilot study. Surg Innov.

[26] Benzoni C, Benini B, Pirozzi C (2004). Gallbladder strangulation within an incisional hernia. Hernia.

[27] Goldman G, Rafael AJ, Hanoch K (1985). Acute acalculous cholecystitis due to an incarcerated epigastric hernia. Postgrad Med J.

[28] Boer J, Boerma D, de Vries Reilingh TS (2011). A gallbladder torsion presenting as acute cholecystitis in an elderly woman: a case report. J Med Case Reports.

[29] Knab LM, Boller AM, Mahvi DM (2014). Cholecystitis. Surg Clin North Am.

[30] Cross AJ, Buchwald PL, Frizelle FA, Eglinton TW (2017). Meta-analysis of prophylactic mesh to prevent parastomal hernia. Br J Surg.

[31] Mäkäräinen-Uhlbäck EJ, Klintrup KH, Vierimaa MT (2020). Prospective, randomized study on the use of prosthetic mesh to prevent a parastomal hernia in a permanent colostomy: results of a long-term follow-up. Dis Colon Rectum.

[32] Hansson BM, Slater NJ, van der Velden AS, Groenewoud HM, Buyne OR, de Hingh IH, Bleichrodt RP (2012). Surgical techniques for parastomal hernia repair: a systematic review of the literature. Ann Surg.

